# Supplementary material for: Causal interplay between lactose intolerance and gut microbiota: a combined bidirectional Mendelian randomization and in vivo validation study
Source: Front Nutr. 2026 Jun 1;13:1803337. doi: 10.3389/fnut.2026.1803337 (PMC13265576; doi:10.3389/fnut.2026.1803337)
Supplement: Supplementary file 1 [file Data_Sheet_1.zip › supplementary materials/Forward/forest plot/ebi-a-GCST90027774.finngen_R12_E4_LACTONAS.pdf]

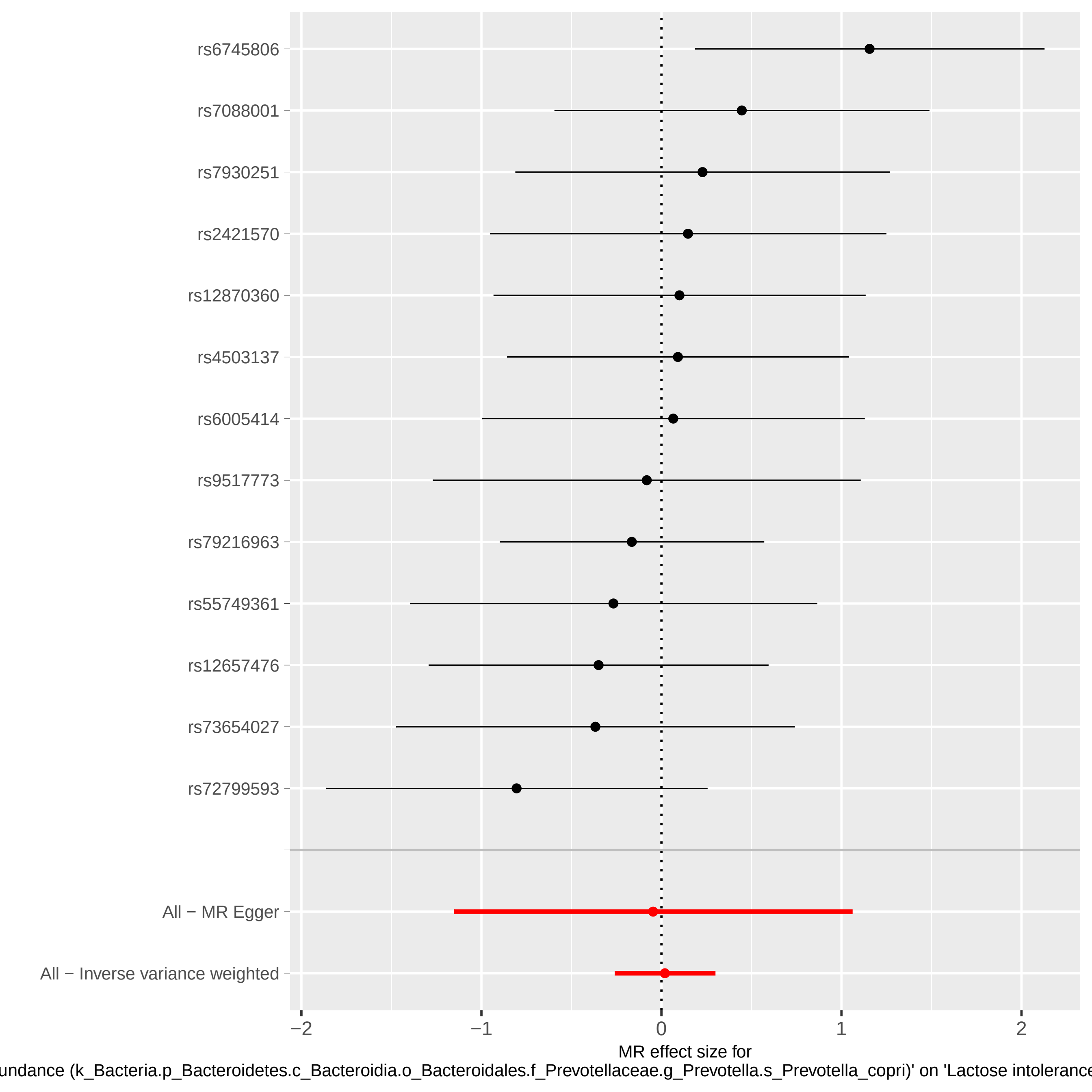
rs6745806
rs7088001
rs7930251
rs2421570
rs12870360
rs4503137
rs6005414
rs9517773
rs79216963
rs55749361
rs12657476
rs73654027
rs72799593
All − MR Egger
All − Inverse variance weighted
−2
−1
0
1
2
MR effect size for
undance (k_Bacteria.p_Bacteroidetes.c_Bacteroidia.o_Bacteroidales.f_Prevotellaceae.g_Prevotella.s_Prevotella_copri)' on 'Lactose intolerance
